# Sodium-Glucose Cotransporter 2 Inhibitors: A Scoping Review of the Positive Implications on Cardiovascular and Renal Health and Dynamics for Clinical Practice

**DOI:** 10.7759/cureus.37310

**Published:** 2023-04-08

**Authors:** Saliha Erdem, Anoop Titus, Dhruvil Patel, Neel N Patel, Yasar Sattar, James Glazier, M. Chadi Alraies

**Affiliations:** 1 Internal Medicine, Wayne State University School of Medicine, Detroit, USA; 2 Internal Medicine, Saint Vincent Hospital, Worcester, USA; 3 Medicine, Government Medical College Thrissur, Thrissur, IND; 4 Internal Medicine, New York Medical College/Landmark Medical Center, Woonsocket, USA; 5 Medicine, B. J. (Byramjee Jeejeebhoy) Medical College, Ahmedabad, IND; 6 Cardiology, West Virginia University, Morgantown, USA; 7 Internal Medicine, Icahn School of Medicine at Mount Sinai, New York City, USA; 8 Cardiology, Wayne State University/Detroit Medical Center, Detroit, USA

**Keywords:** heart failure with preserved ejection fraction (hfpef), hfmref, hfref, sglt-2 inhibitor, heart failure, sodium-glucose cotransporter-2 (sglt2) inhibitors

## Abstract

Cardiorenal benefits of sodium-glucose cotransporter 2 inhibitors (SGLT2is) have been demonstrated in patients with type 2 diabetes in multiple trials. We aim to provide a comprehensive review of the role of SGLT2i in cardiovascular disease. Reducing blood glucose to provide more effective vascular function, lowering the circulating volume, reducing cardiac stress, and preventing pathological cardiac re-modeling and function are the mechanisms implicated in the beneficial cardiovascular effects of SGLT2 inhibitors. Treatment with SGLT2i was associated with a decrease in cardiovascular and all-cause mortality, acute heart failure exacerbation hospitalization, and composite adverse renal outcomes. Improved symptoms, better functional status, and quality of life were also seen in heart failure with reduced ejection fraction (HFrEF), heart failure and mildly reduced ejection fraction (HFmrEF), and heart failure with preserved ejection fraction (HFpEF) patients. Recent trials have shown a notable therapeutic benefit of SGLT2is in acute heart failure and also suggest that SGLT2is have the potential to strengthen recovery after acute myocardial infarction (AMI) in percutaneous coronary Intervention (PCI) patients. The mechanism behind the cardio-metabolic and renal-protective effects of SGLT2i is multifactorial. Adverse events may occur with their usage including increased risk of genital infections, diabetic ketoacidosis, and perhaps limited amputations; however, all of them are preventable. Overall, SGLT2i clearly has many beneficial effects, and the benefits of using SGLT2i by far outweigh the risks.

## Introduction and background

Cardiorenal benefits of sodium-glucose cotransporter 2 inhibitors (SGLT2is) have been demonstrated in large-scale clinical trials in patients with type 2 diabetes and either with an established cardiovascular disease or multiple cardiovascular risk factors [1-3]. In addition, SGLT2i has a favorable metabolic profile as they have been shown to significantly reduce atherosclerotic events, heart failure hospitalization, cardiovascular and total mortality, and chronic kidney disease progression [4]. Given these treatment advantages, this class of medications is now being used in patients without diabetes to treat heart failure and chronic kidney disease [5]. This review provides the current understanding of the practical use of the cardio-metabolic-renal benefits of SGLT2i. It discusses the benefits and side effects of its use in the most common cardiovascular patient profiles by presenting the most up-to-date trials being conducted on the subject. In this review, using evidence-based practice, we will provide a comprehensive review of the role of SGLT2i in cardiovascular disease, particularly in heart failure, and the novel findings in acute myocardial infarction, alongside discussing mechanistic implications of their global benefits on cardiovascular and renal disease and side effects.

SGLT2 is a sodium-glucose cotransporter located within the luminal side of the S1 and S2 regions of the first segment of the proximal convoluted tubule [6]. The SGLT2 class of receptors is divided into subtypes 1 and 2, with 1 being most prevalent within the intestinal lumen while 2 being present within the nephron. Yet both receptors are present in each region [7]. SGLT2 in particular is the primary site for glucose reabsorption within the nephron, where upward of 90% of total urinary glucose reabsorption occurs, specifically an estimated 120-180 g of glucose daily through the coupling of Na+ to glucose to provide ionic neutrality for transport through the tubular membrane at a ratio of 1:1 [8,9]. The remaining 10% of glucose filtered load is managed by the SGLT1 receptors functioning at 2:1 coupling of Na+ to glucose for absorption within the S3 region of the proximal tubule [6,10,11]. Specifically, in either receptor, both complexes travel an inward sodium gradient into tubular cells by way of extravasation of Na+ by Na+/K+ pumps that work to remove Na+ from each cell [12].

Inhibition of the SGLT2 receptor in the kidney specifically propagates limited glucose reabsorption with limited downstream nephron compensation to uptake remaining tubular glucose [13]. This promotes a glycosuric environment within the nephron lumen with an inversely proportional relationship between urine glucose and serum glucose levels thereby being established. Current generation inhibitors are derivatives of phlorizin, a glucoside naturally functioning as a nonselective competitive inhibitor of both SGLT classes of receptors [14]. SGLT2 inhibitors specifically have been derived to possess upward of 1000x more affinity as a competitive receptor inhibitor for SGLT2 compared to SGLT1 [15]. In contrast, phlorizin nonselective receptor properties limited its potential as a therapeutic tool.

Specific molecular interactions between SGLT2 receptors and these inhibitors have not been well delineated, but studies to determine signaling pathways involved have characterized both upstream and downstream cascading relationships from SGLT2 receptor inhibition. Sixteen publicly available dataset molecular models have shown that SIRT6, a silencing factor of the histone deacetylase SIRT1 that normally acts to increase SGLT2 expression, is a notable suppressor of glucose metabolism [16,17]. Both factors are found abundantly in renal tissue and have been hypothesized as potential therapeutic targets for kidney disease [18,19]. The binding of SGLT2 inhibitors may increase SIRT1 expression, causing a downstream SIRT6 increase which may potentiate metabolism inhibition of glucose and prevent further SGLT2 receptors concentration from developing at the tubular region. Furthermore, activation of protein kinase A and protein kinase C has been notably increased when SGLT2 receptors absorb glucose in human embryonic kidney cells, suggesting that SGLT2i may be downregulated downstream signaling cascade related to both these enzymes at the proximal convoluted tubule [20]. Yet, despite current molecular modeling tools, there is minimal evidence to identify the specific mechanism of interactions that cause this inhibition. On a broader profile though, pharmacodynamic and kinetic investigations have noted that both the long-acting and short-acting inhibitors have long retention with broad distribution throughout renal tissue. But in the investigation by Tahara et al. [21], long-acting inhibitors have better stability for blood glucose than their short-acting counterparts, indicating the potential roles of each drug for a variety of patient characteristics, necessitating further investigations.

## Review

Mechanisms implicated in beneficial cardiovascular effects of SGLT2 inhibitors

**Figure 1:**
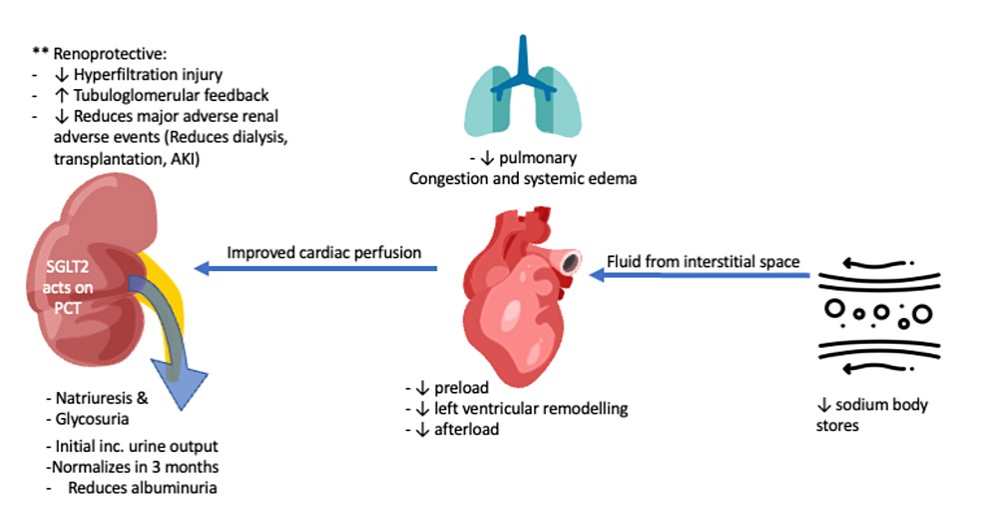
Mechanisms of SGLT-2 inhibitors Image credit: Anoop Titus. AKI: acute kidney injury, SGLT-2: sodium-glucose cotransporter 2.

While there are a wide variety of glucose transporters in cardiac tissue, the most common glucose transporters (GLUT) are GLUT1 and GLUT4, which function through passive transport [22]; there is no evidence for SGLT2-specific receptor expression in cardiac tissue [23]. This has led to the likely possibility that the positive impact of SGLT2i on cardiovascular function is from the indirect effects stemming from glucose-mediated osmotic natriuresis after medication involvement [24] and the particular domains can be grouped along the following points as given in the following sections (Figure 1).

Reduction in Blood Glucose by SGLT2i Promotes More Effective Vascular Function

The increased glucose concentrations in type 2 diabetic patients have been notably linked with worsened vascular dysfunction [25]. Glucose can function as an inflammatory promoter in non-physiological concentrations when interacting with vasculature resulting in tortuous regeneration that negatively impacts vascular dynamics [26]. On a systemic scale, continued exposure may emerge in higher resistance areas that induce end-organ damage and increased cardiac load, potentially promoting increased cardiac stress. SGLT2i has been shown to lower blood pressure by 3-5 mmHg via cardiac afterload reduction without interfering with heart rate and arterial stiffness [2,27]. This reduction in blood pressure is independent of low glomerular filtration rate (GFR) which might suggest that SGLT2i decreases sympathetic nervous activity in heart failure [28]. Both these features can influence the health stabilization of cardiac function when these medications are used.

SGLT2i Lowering Circulating Volume and Reducing Cardiac Stress

Glucose control through diuresis with SGLT2is can impact blood volume. Transient elevations in blood volume after glucose ingestion have been observed in an ingestion study by Evans et al. [29]. When extrapolated, elevated blood glucose can produce a transient elevation in circulating blood volume from baseline which directly necessitates increased cardiac activity to maintain healthy output. The clearance of glucose from SGLT2i can control this transient blood glucose elevation, limiting strenuating cardiac exertion.

As evidence has outlined a therapeutic benefit in chronic heart failure (HF) management, the setting of acute diminished cardiac activity, specifically acute heart failure, has started to garner more focus. In this setting, there remains a persistent excessive fluid accumulation in both the vascular compartment and the interstitial space from inadequate forward pressure causing stasis in susceptible regions due to an acute elevation in cardiac demand causing exhaustion. Traditional diuretic therapy functions to achieve a euvolemic state through electrolyte removal until both interstitium and intravascular volumes are in balance. In contrast, SGLT2is actually have a much more potent osmotic diuresis effect through tubular glucose rather than electrolyte mechanism, potentially making them more effective than the current standard of care. The downstream implications of this are described by Hallow et al. [30]; due to SGLT2i’s water clearance effect being primarily osmotic, there is a resultant elevated fluid clearance from interstitial space as osmotic diuresis causes a transient hypertonic intravascular environment. They hypothesize that this volume difference can affect congestion relief with minimal impact on blood volume and thereby perfusion potential throughout the body, similar to what is achieved by the current diuretic standards of care. An important risk of diuretic use causing an abrupt intravascular volume loss is compensatory reflexive tachycardia, which can overload recovering cardiac tissue during HF exacerbations. The euvolemic diuresis potential of SGLT2 inhibitors can limit this particular risk and likely provide a favorable hemodynamic environment that stabilizes heart function without unnecessary effects. These features have specific downstream implications that can promote a healthy cardiac environment through a reduction in cardiac preload and potentially prevent pulmonary congestion and systemic edema with clinical investigations in acute HF being discussed later.

SGLT2i Has Been Implicated in Preventing Pathological Cardiac Remodeling and Function

Recent evidence has pointed toward a potential anatomical impact of SGLT2i use on heart tissue. Rat models by Joubert et al. [5,31] demonstrate that the implementation of dapagliflozin induced a notable reduction in a lipodystrophy model compared to those untreated that significantly reduced the pathologic process leading to hypertrophic cardiomyopathy. The same drug was also tested in Black and Tan Brachyury (BTBR) mice by Moy et al., which are bred for insulin resistance [32], and was found to limit the fibrotic impact and pathological remodeling of the left ventricle implicating SGLT2i as the potential for improving heart function [33]. Among a type I diabetic rat model induced by streptozotocin, dapagliflozin had a lower type I-to-type III collagen ratio and the most common type of collage found in the myocardium [34] with lesser reactive oxygen species concentrations in tissues [35]. Findings correlate with improved myocardial tissue health and thereby can be associated with a likely potential benefit in reducing harmful cardiac changes with SGLT2i use.

Extending these findings into the clinical setting, a prospective trial conducted by Gamaza-Chulián et al. demonstrates that after the use of SGLT2i, there was an average decrease in left ventricular mass of +2.34 ± 4.13 g/m^2^, significantly more than the control group without prescribed inhibitors, and with an increased global longitudinal strain (on average of 0.29 ± 0.47 (P = 0.011)), denoting these trends as being positive changes [36]. There is also a notable improvement, from a randomized controlled trial conducted by Rau et al., of the left ventricular filling pressure in diastole when empagliflozin was introduced to patients, but this trial did not note any improvement in left ventricular systolic function [37]. These findings suggest not only an anatomical benefit but also related improvement in myocardial functionality; yet, data on these findings are scarce compared to other classes of cardiovascular medications that have also been found to positively impact cardiac remodeling and function, necessitating an avenue for more investigations. More importantly, there are still no direct mechanistic findings published on what pathways are implicated in these phenomena from SGLT2 inhibition requiring delineation before these conclusions can be validated.

Clinically Noticeable Cardiovascular Benefits of SGLT2 Inhibitors

Many randomized clinical trials initially showed that SGLT2i decreases mortality, heart failure hospitalization, and all-cause death in patients with type 2 diabetes mellitus (DM2) who are at high risk [1-3,38-41]. The majority of the patients in these trials did not have heart failure, and even if they did, it was not well characterized. About 30% of the reduction was seen in heart failure hospitalization, and the effect seemed to be consistent across all the SGLT2i classes.

DAPA-HF (Study to Evaluate the Effect of Dapagliflozin on the Incidence of Worsening HF or Cardiovascular Death in Patients with Chronic HF) and the EMPEROR-Reduced (Cardiovascular and Renal Outcomes with Empagliflozin in Heart Failure) trials recently demonstrated the benefit of SGLT2i in patients with established heart failure with reduced ejection fraction [39,40,42]. Both trials enrolled patients with and without diabetes mellitus who were receiving standard therapy for heart failure. In addition, a recent meta-analysis [43] of these two trials has shown that when SGLT2i (empagliflozin or dapagliflozin) is added to guideline-directed medical therapy for the treatment of heart failure with reduced ejection fraction, there is a decrease in cardiovascular and all-cause mortality, acute heart failure exacerbation hospitalization, and composite adverse renal outcomes regardless of the disease severity. The heterogeneity of effect size was not significant between the trials. The meta-analysis characterized SGLT2i effects in relevant subgroups and showed consistent benefits (decrease in the combined risk of cardiovascular death or hospitalization for heart failure) in subgroups that were based on type 2 diabetes status, age, sex, angiotensin receptor-neprilysin inhibitor (ARNI) treatment, history of hospitalization for heart failure, estimated baseline glomerular filtration rate (eGFR), and body mass index. There was no proof of treatment-by-subgroup interaction for each subgroup except for those of subgroups based on New York Heart Association (NYHA) functional class and race (pooled hazard ratio (HR): 0.67, 95% confidence interval (CI): 0.59-0.76 for NYHA class 2 vs HR: 0.87, CI: 0.75-1.01 for NYHA classes 3-4). Treatment with SGLT2i was also associated with improved symptoms, better functional status, and quality of life in patients with heart failure with reduced ejection fraction.

Another meta-analysis [44] of six clinical trials comparing the effect of ARNI vs SGLT2i in the treatment of heart failure with reduced ejection fraction (HFrEF) confirmed the findings of the aforementioned studies. The study demonstrated that SGLT2i significantly reduces the risk of cardiovascular death, hospitalization for heart failure, and all-cause mortality and has even better performance when combined with ARNI. The reduction in cardiovascular death or hospitalization for heart failure was independent of underlying diabetes mellitus in patients on SGLT2i.

Surprisingly, these medications also slowed the progression of kidney disease. There is an observed reduction in creatinine, albuminuria, and mortality attributable to renal disease when these medications are started [45,46]. These protective effects seem to be independent of anti-glycemic effects. In light of these encouraging findings, SGLT2i is extremely suggested for patients with type 2 diabetes who are at high cardiovascular and renal risks.

SGLT2i and Heart Failure With Preserved Ejection Fraction (HFpEF)

While additional trials are ongoing for use of SGLT2i in patients with heart failure with preserved ejection fraction (HFpEF; left ventricular ejection fraction (LVEF): ≥50%), the EMPEROR-Preserved trial [42] tested the beneficial effect of SGLT2i in these patient populations. The trial showed that compared to a placebo, empagliflozin decreases the combined risk of cardiovascular death or hospitalization for heart failure in patients with HFpEF regardless of diabetes status. The trial included patients with an ejection fraction greater than 40% and NYHA classes 2-4. Therefore, the conclusion of this trial can be applied to patients with heart failure and mildly reduced ejection fraction (HFmrEF; LVEF: 41-49%). In the recently presented phase 3 Dapagliflozin Evaluation to Improve the Lives of Patients with Preserved Ejection Fraction Heart Failure (DELIVER) trials among 3,131 patients with HFpEF who received dapagliflozin compared to 3,132 patients in the control group, there was a notable reduction in heart failure hospitalizations [47]. Furthermore, mortality was also reduced, serving as another landmark trial recently published to provide further evidence of the therapeutic benefit of SGLT2i in patients with HFpEF.

SGLT2i and Acute Heart Failure

An emerging area of research for SGLT2is is their use in the treatment of acute heart failure. Recently published randomized controlled trials have begun to show a notable therapeutic benefit of SGLT2is in acute heart failure settings among all patients, including those with type 2 diabetes mellitus [48]. In the double-blinded, placebo-controlled, randomized controlled trials, Empagliflozin in Patients Hospitalized for Acute Heart Failure (EMPULSE) trials, there were 265 patients receiving 10 mg of empagliflozin during an acute HF exacerbation compared to the same number of controls receiving a placebo. The trial demonstrated lower inpatient mortality and with a more favorable win ratio (stratified win ratio, 1.36; 95% confidence interval, 1.09-1.68; P = 0.0054), a composite of mortality of any cause, the incidence of HF events and time to first heart failure event, or a 5 point or greater difference in change from baseline in the Kansas City Cardiomyopathy Questionnaire Total Symptom Score at 90-day follow-up. This benefit was evidenced across all ejection fractions and in both denovo and chronic HF patients [49]. These results were most recently followed by the Effects of Empagliflozin on Clinical Outcomes in Patients With Acute Decompensated Heart Failure (EMPA-RESPONSE-AHF) trial. In their randomized, double-blind, parallel-group trial of 41 patients receiving empagliflozin compared to 39 control groups receiving a placebo, there was a combined endpoint reduction of in-hospital HF worsening and rehospitalization for HF or death at 60 days in those receiving empagliflozin compared to the placebo (4 (10%) vs. 13 (33%); P = 0.014). There was also notably no significant difference in the length of stay for patients receiving either intervention [50]. Acute heart failure interventions are complex and often have varying sensitivities across patients, but the results that SGLT2i implements into the most common protocols may have wide adaptability.

SGLT2i Use in Acute Myocardial Infarction Management

Recently, due to the global effects of SGLT2i to reduce cardiac stress in HF being validated, there has also been attention placed on the benefits of medication during acute myocardial infarction management and recovery [51]. Animal models on these effects show contrasting results. In a study [38] looking at the protective effects of SGLT2i in the setting of ischemia followed by reperfusion, Andreadou et al. initially fed mice with empagliflozin for six weeks and then ligated the left anterior descending (LAD) coronary artery for 30 minutes followed by two hours of reperfusion period. Empagliflozin pretreatment was found to be associated with reduced infarct size by approximately 50% and improved left ventricular fractional shortening from 41% to 44% compared to control animals. Asensio Lopez et al. [52] observed similar results following permanent ligation of the LAD. Surprisingly, another study [53] demonstrated that myocardial infarction (MI)-induced acute kidney injury (AKI) was also attenuated in animals pretreated with empagliflozin. In contrast, other animal studies fail to show similar benefits (reduced infarction size) when SGLT2i was used shortly before or during MI [54,55]. Likewise, treatment initiation following the ischemic event did not provide any benefits [56,57].

The recently published Empagliflozin in Acute Myocardial Infarction (EMMY) trial, a multi-center, double-blind trial compared the outcomes of 237 patients with an acute myocardial infarction receiving 10 mg once daily of empagliflozin with 239 patients receiving placebo within three days after percutaneous coronary intervention. There was a follow-up period of 26 weeks where a notable primary outcome of a reduction in N-terminal pro-B-type natriuretic peptide (NT-proBNP) was significantly greater in the intervention group (15% lower, 95% confidence interval (CI): 4.4% to −23.6%) alongside the absolute LV ejection fraction also significantly improving with those receiving empagliflozin (1.5%, 95% CI: 0.2-2.9%, P = 0.029) [58]. This suggests that SGLT2is have the potential to strengthen recovery after acute myocardial infarction (AMI) in percutaneous coronary intervention (PCI) patients and encourages investigations to be conducted among myocardial infarction patients.

Renoprotective Associations of SGLT2 Inhibitors

In addition to their cardiovascular benefits, SGLT2i also has renoprotective effects. CANVAS-R [1] was the first trial that reported these protective effects. The trial demonstrated that canagliflozin reduced the progression of albuminuria, the risk of the composite outcome of a sustained 40% reduction in eGFR, the need for renal replacement therapy, or death from renal causes in patients with diabetic kidney disease. Later, both DAPA-CKD and CREDENCE trials have shown that the patients treated with SGLT2i had less of a composite sustained decline in estimated GFR of at least 50%, end-stage kidney disease, or death from renal or cardiovascular causes compared to placebo. The DAPA-CKD demonstrated these favorable effects in both patients with diabetes vs those without. Furthermore, these outcomes were also confirmed by other cardiovascular outcomes trials [5,41,59,60]. A pooled analysis of these four trials indicated that SGLT2i reduces major renal adverse events including dialysis, transplantation, or mortality due to kidney disease and protects against acute kidney injury [61]. Furthermore, secondary analysis of the EMPA-REG OUTCOME trial revealed that the severity of chronic kidney disease (CKD) or the presence of HF did not modify the favorable effects on kidney outcomes [62,63].

Although the glucose-lowering effect diminishes in patients with CKD, renal benefits of SGLT2i persist. Therefore, the current eGFR limitation put by FDA (eGFR ≥45 ml/min/1.73 m^2^ ) should be reconsidered [13] as cardiorenal benefits occur independently of glucosuria, based on findings observed in DAPA-HF trial in patients without diabetes [40]. Empagliflozin treatment in diabetic kidney disease mouse models was also conducted by Lu et al. [64]. In the extraction of renal tissue, both proteomic and metabolomic analyses subsequently done demonstrated a positive alteration in renal tissue by preventing glomerular hypertrophy to the extent that the diabetic controlled had possessed after 12 weeks alongside a substantial decrease in interstitial fibrosis (at 62.8% compared to 68.5%). There was also a notable difference in protein excretion with a reduced amount in the intervention arm. When all these data are taken together, it is reasonable to prescribe SGLT2i to patients with CKD irrespective of the presence of diabetes. This evidence is in line with recent reports published that can conclude that SGLT2i not only has a role in preventing chronic cardiovascular complications but also in the mitigating progression of chronic renal disease [65].

The mechanism behind these positive effects is multifactorial and might be due to the activation of tubuloglomerular feedback, limiting the hyperfiltration injury, reduction in glomerular hypertension and kidney hypoxia, and likely effects on sodium-hydrogen exchange [5,66]. Furthermore, the improvements in cardiac function might be responsible for their benefits on the renal outcome by halting the “vicious cardiorenal circle” [5,67]. Like other diuretics though, the SGLT2 diuretic effect diminishes over time due to compensatory mechanisms that achieve a stable state. Although they are responsible for increased urine output, this effect normalizes following three months of treatment initiation [68,69] and necessitates more nuanced investigations to provide an accurate profile of medication effects and benefits through the lens of this mechanism.

National and International Guidelines

The 2021 clinical practice guidelines by the European Society of Cardiology (ESC) for the diagnosis and treatment of acute and chronic heart failure recommend glucagon-like peptide-1 receptor agonists (GLP-1 RAs) or SGLT2i in diabetic patients with established atherosclerotic cardiovascular diseases (ASCVDs) and/or target organ damage (TOD) to decrease cardiovascular events (class 1). Similarly, SGLT2i is suggested as a class 1 recommendation in diabetic patients with HFrEF on top of standard therapy or CKD to decrease cardiovascular death and heart failure hospitalization and improve ASVD and cardiorenal outcomes [70].

The American Diabetes Association guidelines recommended SGLT2i as a first-line treatment in patients with diabetes and HF or at high risk of HF [71]. Per the 2019 version of Management of Hyperglycemia in Type 2 Diabetes, a consensus report by the American Diabetes Association and the European Association for the Study of Diabetes, SGLT2i or GLP-1 RAs can be started in the appropriate high-risk individuals with established type 2 diabetes to decrease heart failure hospitalization, major adverse cardiovascular events (MACE), cardiovascular (CV) death, or CKD progression independent of baseline hemoglobin A1c (Hba1c) [41]. Treatment is most beneficial in patients with or without established atherosclerotic cardiovascular disease but with HFrEF (left ventricular ejection fraction (LVEF) < 45%) or CKD (eGFR 30-60 mL/min per 1.73 m^2^ or urine albumin:creatinine ratio (UACR) > 30 mg/g, particularly when UACR ≥ 300 mg/g).

Many guidelines do not address patients without type 2 diabetes. However, the 2022 American Heart Association/American College of Cardiology/Heart Failure Society of America (AHA/ACC/HFSA) guidelines recognize SGLT2i as a part of four classes of medications for the treatment of HFrEF and recommend it as a class 1a evidence in the treatment of HFrEF irrespective of the presence of type 2 diabetes [72]. Likewise, they are suggested as class 2a evidence for HFmrEF (symptomatic HF with LVEF: 41%-49%). Similar to AHA/ACC, the Canadian Cardiovascular Society/Canadian Heart Failure Society’s guidelines recommended the prescription of SGLT2 in patients with HFrEF (LVEF < 40%) and without coexisting diabetes to reduce the risk of hospitalization and cardiovascular mortality [73].

Safety

SGLT2i is associated with a higher risk of genital infections in both genders, although they do not increase the risk of urinary tract infections, including pyelonephritis [4,74]. These infections generally are not dangerous and tend to resolve following brief treatment with antifungal therapy. Fournier gangrene is a rare but feared complication of SGLT2i; however, more recent trials have not confirmed it [2]. Like any other diuretics, they can cause volume depletion, but no increased risk of acute kidney injury has been reported.

Although rare, SGLT2i has been shown to cause euglycemic diabetic ketoacidosis (DKA) so it is important to measure ketone levels in suspected patients [47]. Four large clinical trials including CREDENCE reported 74 events in 38,702 patients (0.2%), so the incidence is quite low [4,7]. In fact, a recent meta-analysis did not increase the risk of DKA in patients taking SGLT2i compared to placebo [49].

In addition, the CANVAS program reported a higher rate of fractures and amputations in patients treated with canagliflozin, especially at the toe and metatarsal level [1]; however, other clinical trials (EMPA-REG OUTCOME and DECLARE-TIMI 58) did not show a significantly increased risk of these fractures. Importantly, the CREDENCE trial also did not report a higher incidence of fractures with canagliflozin use [74]. However, caution should be taken in patients with pre-existing peripheral arterial disease and/or lower extremity ulcers as the risk of fractures cannot totally be excluded [75].

Currently, there are two SGLT2is, both empagliflozin and dapagliflozin, that show a cardiovascular benefit post-phase 3 trials, particularly for patients with HFpEF, who were previously medically managed through a complex regimen [40,42,47]. Yet, the evidence supporting both empagliflozin and dapagliflozin has translated into an ongoing trial to evaluate the ideal regimen of both drugs, 10 mg each, for acute/decompensated HF medical management. This is currently being studied in the EMPATHY (Empagliflozin and Dapagliflozin in Patients Hospitalization for Acute Decompensated Heart Failure) trial [76]. The prominent clinical trials on SGLT2i are shown in Appendix, Table 1.

Expert Opinion

This review outlines the surmounting evidence for SGLT2 inhibitor use in preventing both renal and cardiac diseases. These features have opened new routes of investigations into chronic cardiovascular comorbidity management and assist in positively augmenting cardiovascular recovery. With SGLT2 inhibitors being validated as a new class of drugs for treating HFpEF, the emergence of these drugs being used across all spectrums of heart failure has created reinvigorated interest in stratifying benefits among this group of patients. The clinical benefits have been validated in large randomized controlled trials; yet, the specific mechanisms of these effects are only still hypothetical and remain a point of study. Furthermore, the management of acute heart failure exacerbations has remained unchanged, but the introduction of SGLT2 inhibitors may emerge as a welcome addition. Recently published trials showed an improved hospitalization course, reduced readmission risk, and most importantly improved mortality. These findings lay the groundwork for SGLT2 inhibitors to be recommended for acute heart failure management.

Management of acute myocardial infarction and recovery from any intervention have primarily been focused on preventing vascular complications. SGLT2 inhibitors have been observed to drastically improve recovery rates that have been tracked by surrogate biomarkers, including NT-proBNP, evidencing facilitation toward native function occurring. Hypothetically, a reduction in hormonal and volume stress emerges from SGLT2 inhibitors which further stimulate a pro-recovery environment. Mouse models have also shown a reduction in adverse cardiac remodeling after infarction models which may indicate a direct anatomical mechanism of these drugs [51]. Prevention of complications post-PCI has also emerged as a paradigm of study. The published EMMY trial has provided evidence of SGLT2 inhibitors being introduced into the medical management regimen in PCI patients [57].

Recovery, and reversibility, of renal injury has also been a clinically advantageous phenomenon of SGLT2 inhibitor use. Prevention of further kidney disease, being observed consistently across all spectrums of CKD, concomitantly has also reduced the risk of further cardiovascular stress emerging from compensatory mechanisms. Finally, it is important to recognize that with strong evidence for SGLT2 inhibitors producing a positive benefit in both acute and chronic cardiovascular pathologies, the introduction of SGLT2 inhibitors into medical regimens for these pathologies is likely going to emerge.

## Conclusions

SGLT2i clearly has many beneficial effects beyond its glucose-lowering effect. Many large-scale clinical trials have shown that they reduce cardiovascular mortality, all-cause mortality, and the progression of CKD along with reducing heart failure hospitalization and atherosclerotic events. The drug class is now even recommended in patients with HFrEF and/or CKD independent of the presence of concomitant diabetes. More studies are ongoing for its use in acute decompensated heart failure and heart failure with a preserved ejection fraction. The mechanism behind the cardio-metabolic and renoprotective effects is multifactorial. Adverse events may occur with their usage including increased risk of genital infections, diabetic ketoacidosis, and perhaps limited amputations; however, all of them are preventable. The benefits obviously outweigh the risks, which should give cardiologists enough confidence to prescribe them.

## Appendices

**Table 1:** Prominent clinical trials on SGLT2i SGLT2i: sodium-glucose cotransporter 2 inhibitor, HF: heart failure, DM: diabetes mellitus, CV: cardiovascular, KCCQ: Kansas City Cardiomyopathy Questionnaire, eGFR: estimated baseline glomerular filtration rate, NYHA: New York Heart Association, LVEF: left ventricular ejection fraction, UACR: urine albumin to creatinine ratio, MI: myocardial infarction, NT-proBNP: N-terminal pro-B-type natriuretic peptide, TSS: Total Symptom Score, ESRD: end-stage renal disease, T2DM: type 2 diabetes mellitus, SGLT2: sodium-glucose cotransporter 2, CKD-EPI: Chronic Kidney Disease Epidemiology Collaboration, Cr: creatinine, HFrEF: heart failure with reduced ejection fraction, ACE-I: angiotensin-converting enzyme inhibitor, ARB: angiotensin receptor blocker, CVD: cardiovascular death, HbA1c: hemoglobin A1C, AE: adverse events, PCI: percutaneous coronary intervention, LVESV: left ventricular end-systolic volume, LVEDV: left ventricular end-diastolic volume, E/e': peak E-wave velocity/peak e' velocity.

Trial Abbreviation	Trial Name	Inclusion Criteria	Intervention Arm	Control Arm	Primary Outcome	Secondary Outcome	Result
DAPA-HF [40]	Study to Evaluate the Effect of Dapagliflozin on the Incidence of Worsening HF or Cardiovascular Death in Patients With Chronic HF	Patients with heart failure with reduced ejection fraction and with and without DM	Dapagliflozin 5 or 10 mg	Placebo matching dapagliflozin of same dose	1. CV death, hospitalization due to heart failure or urgent visit due to heart failure.	1. Subjects included in the composite endpoint of CV death or hospitalization due to heart failure. 2. Events included in the composite endpoint of recurrent hospitalizations due to heart failure and CV death. 3. Change from baseline in the KCCQ total symptom score. 4. Subjects included in the composite endpoint of ≥50% sustained decline in eGFR, ESRD, or renal death. 5. Subjects included in the endpoint of all-cause mortality.	Among individuals with HFrEF (NYHA II-IV, LVEF ≤40%) with or without T2DM, addition of SGLT-2 inhibitor dapagliflozin decreased rates of CV death or worsening HF, as well as all-cause mortality.
EMPEROR-Reduced [77]	Empagliflozin Outcome Trial in Patients With Chronic Heart Failure With Reduced Ejection Fraction	Patients with heart failure with reduced ejection fraction and with and without DM	Empagliflozin once daily	Placebo	1. Occurrence of adjudicated hospitalization for heart failure (HHF). 2. eGFR (CKD-EPI) Cr slope of change from baseline. 3. Time to first event in composite renal endpoint: chronic dialysis, renal transplant, or sustained reduction of eGFR (CKD-EPI) cr. 4. Time to first adjudicated hospitalization for heart failure (HHF). 5. Time to adjudicated cardiovascular (CV) death. 6. Time to all-cause mortality. 7. Time to onset of diabetes mellitus (DM). 8. Change from baseline in KCCQ.	1. Occurrence of adjudicated hospitalization for heart failure (HHF). 2. eGFR (CKD-EPI) Cr slope of change from baseline. 3. Time to first event in composite renal endpoint: chronic dialysis, renal transplant, or sustained reduction of eGFR (CKD-EPI) cr. 4. Time to first adjudicated hospitalization for heart failure (HHF). 5. Time to adjudicated cardiovascular (CV) death. 6. Time to all-cause mortality. 7. Time to onset of diabetes mellitus (DM). 8. Change from baseline in KCCQ. 9. Number of all-cause hospitalizations (first and recurrent).	In patients with HFrEF on guideline-directed medical therapy, irrespective of whether T2DM is present or not, the addition of empagliflozin reduces cardiovascular mortality and hospitalization for progression of HF.
EMPEROR-Preserved [42]	Empagliflozin Outcome Trial in Patients With Chronic Heart Failure With Preserved Ejection Fraction	Patients with heart failure with preserved ejection fraction and with and without DM	Empagliflozin 10 mg	Placebo	1. Time to first event of adjudicated cardiovascular (CV) death or adjudicated hospitalization for heart failure (HHF).	1. Occurrence of adjudicated hospitalization for heart failure (HHF). 2. eGFR (CKD-EPI) Cr slope of change from baseline. 3. Time to first event in composite renal endpoint: chronic dialysis, renal transplant, or sustained reduction of eGFR (CKD-EPI) cr. 4. Time to first adjudicated hospitalization for heart failure (HHF). 5. Time to adjudicated cardiovascular (CV) death. 6. Time to all-cause mortality. 7. Time to onset of diabetes mellitus (DM). 8. Change from baseline in KCCQ. 9. Number of all-cause hospitalizations (first and recurrent).	Among adults with heart failure with mid-range or preserved ejection fraction (LVEF >40%), empagliflozin decreased the risk of the cardiovascular death or heart failure hospitalization. This benefit was mainly driven by fewer heart failure hospitalizations.
CANVAS-R [1]	A Study of the Effects of Canagliflozin on Renal Endpoints in Adult Participants With Type 2 Diabetes Mellitus	Must have inadequate diabetes control (as defined by glycosylated hemoglobin level ≥7.0% to ≤10.5% at screening) ≥30 years old with history of cardiovascular (CV) event, or ≥50 years old with high risk of CV events. Must be either not on antihyperglycemic agents (AHA) therapy, or on AHA monotherapy, or combination of AHA therapy with any approved agent for the control of blood glucose levels.	Canagliflozin 100 mg once daily during the first 13 weeks, then the dose may be increased to 300 mg once daily.	Placebo	1. Progression of albuminuria.	1. Composite of cardiovascular (CV) death events or hospitalization for heart failure. 2. Cardiovascular (CV) death.	Among patients with T2DM at high risk for CV events, canagliflozin had a lower risk of cardiovascular events and reduced the rate of renal decline and heart failure hospitalization compared to those who received placebo but a greater risk of amputation.
DAPA-CKD [39]	A Study to Evaluate the Effect of Dapagliflozin on Renal Outcomes and Cardiovascular Mortality in Patients With Chronic Kidney Disease	Aged ≥18 years; eGFR ≥25 and ≤75 mL/min/1.73 m^2^ (CKD-EPI formula) at visit 1; evidence of increased albuminuria three months or more before visit 1 and UACR ≥200 and ≤5,000 mg/g at visit 1 stable, and for the patient maximum tolerated labeled daily dose, treatment with ACE-I or ARB for at least four weeks before visit 1, if not medically contraindicated,	Dapagliflozin 5 or 10 mg	Placebo matching dapagliflozin of same dose	1. Time to the first occurrence of any of the components of the composite: ≥50% sustained decline in eGFR or reaching ESRD or CV death or renal death.	1. Time to the first occurrence of any of the components of the composite: ≥50% sustained decline in eGFR or reaching ESRD or renal death. 2. Time to the first occurrence of either of the components of the composite: CV death or hospitalization for heart failure. 3. Time to death from any cause.	In patients with chronic kidney disease characterized by eGFR reduction and microalbuminuria with and without type 2 diabetes, treatment with dapagliflozin was associated with less progression of CKD, renal mortality, or CVD mortality, when compared to placebo. Of note, there was also a reduction in all-cause mortality with dapagliflozin, but it was not a primary endpoint.
CREDENCE [41]	Evaluation of the Effects of Canagliflozin on Renal and Cardiovascular Outcomes in Participants With Diabetic Nephropathy	Type 2 diabetes mellitus with a HbA1c ≥ 6.5% and ≤ 12.0%; eGFR ≥ 30 mL/min/1.73 m^2^ and < 90 mL/min/1.73 m^2^; participants need to be on a stable maximum tolerated labeled daily dose of an ACE-I or ARB for at least four weeks prior to randomization; must have a urine albumin to creatinine ratio (UACR) of > 300 mg/ g and ≤5000 mg/g	One 100 mg over-encapsulated tablet orally once daily	One matching placebo capsule orally (by mouth) once daily	1. One matching placebo capsule orally (by mouth) once daily.	1. Composite endpoint of CV death and hospitalized heart failure (HHF). 2. Major adverse cardiac event (MACE). 3. Hospitalized heart failure (HHF). 4. Renal composite endpoint. 5. Cardiovascular (CV) death. 6. All-cause mortality. 7. CV composite endpoint.	Among patients with T2DM and diabetic nephropathy characterized by albuminuria and no more than a mild reduction in GFR, canagliflozin 100 mg/d reduces the risk of the composite endpoint of end-stage kidney disease, doubling of serum creatinine from baseline, and death from renal or cardiovascular disease when compared to placebo (HR: 0.70; 95% CI: 0.59-0.82; P = 0.00001).
EMPA-REG OUTCOME [3]	Cardiovascular Outcome Event Trial in Type 2 Diabetes Mellitus Patients	Diagnosis of type 2 DM; age ≥ 18 years patients on diet and exercise regimen who are drug naive or pre-treated with any background therapy. Antidiabetic therapy has to be unchanged for 12 weeks prior to randomization; HbA1c of ≥ 7.0% and ≤ 10% for patients on background therapy or HbA1c ≥ 7.0% and ≤ 9.0% for drug-naive patients; body mass index ≤ 45 at visit 1; high cardiovascular risk	1. High-dose empagliflozin daily 2. Low-dose empagliflozin daily	Placebo	Time to the first occurrence of any of the following adjudicated components of the primary composite endpoint (three-point MACE): CV death (including fatal stroke and fatal MI), non-fatal MI (excluding silent MI), and non-fatal stroke.	1. Percentage of participants with the composite of all events adjudicated (four-point MACE): CV death (including fatal stroke and fatal MI), non-fatal MI (excluding silent MI), non-fatal stroke, and hospitalization for unstable angina pectoris. 2. Percentage of participants with silent MI. 3. Percentage of participants with heart failure requiring hospitalization (adjudicated). 4. Percentage of participants with new-onset albuminuria. 5. Percentage of participants with new-onset macroalbuminuria. 6. Percentage of participants with the composite microvascular outcome.	In this industry-sponsored trial, empagliflozin reduced the rate of CV events and slowed the progression of kidney disease among patients with T2DM and high CV risk.
DECLARE-TIMI 58 [2]	Multicenter Trial to Evaluate the Effect of Dapagliflozin on the Incidence of Cardiovascular Events	Aged ≥40 years; diagnosed with type 2 diabetes; high risk for cardiovascular events	Dapagliflozin + standard of care therapy for type 2 diabetes and for comorbidities and cardiovascular risk factors	Placebo + standard of care therapy for type 2 DM and for comorbidities and cardiovascular risk factors	1. Subjects included in the composite endpoint of CV death, MI, or ischemic stroke. 2. Subjects included in the composite endpoint of CV death or hospitalization due to heart failure.	1. Subjects included in the renal composite endpoint: confirmed sustained ≥40% decrease in eGFR to eGFR <60 ml/min/1.73 m^2^ and/or ESRD and/or renal or CV death. 2. Subjects included in the endpoint of all-cause mortality.	In patients with type 2 diabetes and risk of cardiovascular disease, the use of dapagliflozin did not significantly reduce the risk of MACE. Dapagliflozin did result in lower hospitalization rates due to heart failure.
EMPULSE [49]	Empagliflozin in Patient Hospitalized for Acute Heart Failure	Patients admitted for acute heart failure. Systolic blood pressure >100 mmHg and no symptoms of hypotension for six hours. No increase in standard meditation intervention for six hours. Biomarker signs of acute heart failure (N-terminal pro-B-type natriuretic peptide (NT-proBNP) ≥1600 pg/ml or BNP ≥400 pg/ml during hospitalization or within 72 hours prior to admission).	Empagliflozin 10 mg once a day	Placebo + current standard of care therapy for acute heart failure exacerbation inpatient management	1. A win ratio that was a composite endpoint of all-cause death, number of HF events (including hospitalizations for HF, urgent HF visits, and unplanned outpatient visits), time to first HF event, ≥5 point difference change after baseline from the Kansas City Cardiomyopathy Questionnaire score (KCCQ-TSS) for a 90-day post-treatment follow-up period.	1. Secondary outcomes were each separated component of the win ratio: mortality heart failure events, overall change from baseline in the KCCQ-TSS, acute renal failure events, and body weight change after drug implementation.	There was an overall clinical benefit, defined by positive changes in the overall win ratio, in for those who had empagliflozin compared to those who did not with a significant reduction in mortality, readmissions, and positive changes in quality of life based on the KCCQ-TSS questionnaire.
EMPA-RESPONSE-AHF [50]	The Effects of Empagliflozin on Clinical Outcomes in Patients With Acute Decompensated Heart Failure	Patients >18 years of age hospitalized for acute HF exacerbation with or without diabetes. Estimated GFR >30 mL/min/1.73 m^2^ . Treated with loop diuretics as initial intervention and prior to screening	Receiving empagliflozin 10 mg/day	Placebo + HF interventions based on protocol	1. Change in visual analogue scale (VAS) dyspnea score. 2. The diuretic response (weight change per 40 mg furosemide). 3. The change in N-terminal pro-brain natriuretic peptide (NT-proBNP). 4. Assessing the overall difference in the length of stay.	1. Any signs of worsening of heart failure in clinical status evaluation (defined as worsening signs and/or symptoms of HF that require an intensification of intravenous therapy for HF or mechanical ventilatory, renal or circulatory support). 2. Events of all-cause death and/or HF readmission through day 30 and through day 60 as part of AE assessment.	The combined endpoint of in-hospital worsening HF, rehospitalization for HF, or death at 60 days was reduced in those receiving empagliflozin 10 mg compared to those who received the placebo.
EMMY [58]	Empagliflozin in Patients With Acute Myocardial Infarction	Patients with large myocardial infarction, accompanied with large creatine kinase of >800 IU/L receiving PCI within 72 hours of diagnosis. Troponin level 10x upper limit of normal.	Receiving empagliflozin 10 mg/day	Placebo	1. NT-proBNP change over 26 weeks after intervention begins.	1. Echocardiographic parameters including LVEF, LVESV, LVEDV, and E/e’ ratio.	1. 15% reduction in NT-proBNP in the intervention group with improvement in LV functionality and volume identified through echocardiography.
